# Does adaption require a complex symphony or just “three chords and the truth?”

**DOI:** 10.1371/journal.pbio.3003679

**Published:** 2026-03-27

**Authors:** Klas I. Udekwu, Christopher J. Marx

**Affiliations:** Department of Biological Sciences, University of Idaho, Moscow, Idaho, United States of America

## Abstract

How predictable are the collateral effects of adaptation? This Primer explores a new PLOS Biology study of evolved yeast strains suggesting that growth across environments is fairly predictable because the selected mutations only affected a few latent fitness-impacting phenotypes.

## Teaser

How predictable are the collateral effects of adaptation? A new study of evolved yeast strains published in *PLOS Biology* suggests that growth across environments is fairly predictable because the selected mutations only affected a few latent fitness-impacting phenotypes.

Cellular growth requires the concerted action of thousands of genes, but it is unclear how many of these play a significant role in adaptation, or how predictable their fitness effects should be. One broad type of evolutionary prediction that has emerged is ‘global epistasis,’ a generic trend of diminishing returns for the selective advantage of beneficial mutations when introduced into more fit backgrounds and tested in their original selective environment [1,2]. A very different facet of adaptation one may seek to predict is how selected mutations impact other phenotypes (i.e., pleiotropy), such as growth in alternative environments. Without selection in response to novel perturbations, the pattern of pleiotropy might be expected to be more random and predictions would fall to pieces. Perhaps, however, if there were only a limited number of major cellular processes critical to fitness in a particular selective environment, the patterns for their performance across other environments would simply be dictated by which process was originally impacted.

**Fig 1:**
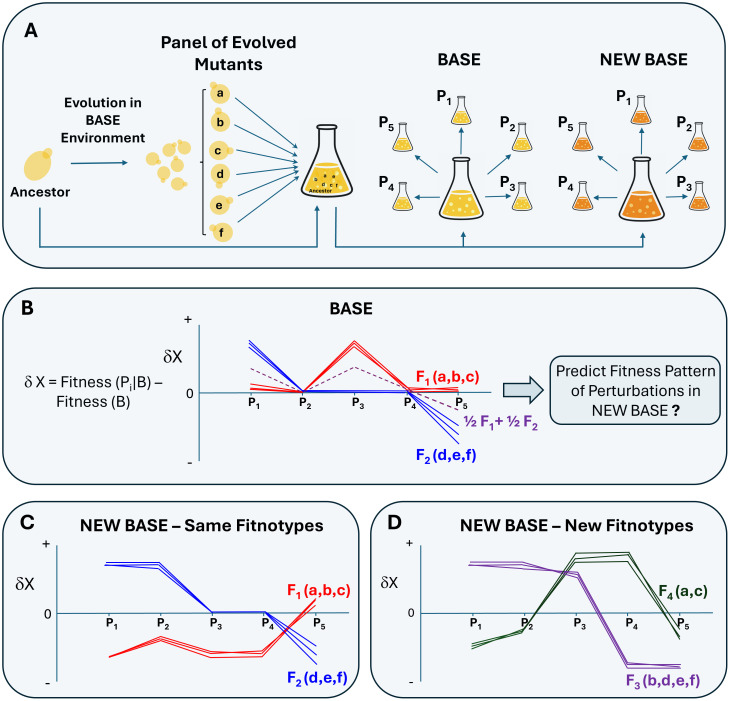
Phenotypic correlations across environments suggest beneficial mutations impact relatively few latent phenotypes underlying fitness. **(A)** A library of 4,000 barcoded, evolved yeast strains was competed simultaneously against their ancestor across a panel of environments (multiple “base” media with individual small perturbations like extra glucose or EtOH). **(B)** The reaction norm of fitness (δX) of evolved yeast strains (a to f) is plotted across a panel of perturbations (P_i_). Each line connects the phenotypes from a single mutant representing its pleiotropic consequence across environments. The phenotypic profile of mutants clustered into discrete patterns (here termed ‘fitnotypes’). For simplicity, two such clusters are shown representing single fitnotypes (red = mutants a, b, c; blue = d, e, f). For example, F_1_ includes mutants who only demonstrated a benefit in P_3_. Note mutant phenotypes can also form linear combinations of fitnotypes (purple). When these mutants are tested across the same perturbations but in a new base environment, the outcome could range, at its extremes, from **(C)** being maintained as the exact same groups that fell into the original fitnotypes, or **(D)** these groups could split, clustering into entirely new sets (purple = b, d, e, f; green = a, c). Data from Ghosh and colleagues [3] indicate that evolved strains fall in between, representing a blend of panels C and D. There remain just three or four fitnotypes per environment, but these still overlap partially with the original fitnotypes.

The Ghosh and colleagues paper in this issue of *PLOS Biology* [3] used a broad panel of barcoded yeast mutants selected for in a “base” environment to ask this question (Fig 1A). They tested the fitness of these evolved strains against their ancestor across a series of perturbations, then using singular value decomposition they asked how correlated the emergent fitness patterns were. In their formulation, fitness in any given environment is the sum of mutational effects upon one of a limited number of key phenotypes (here termed ‘fitnotypes’) multiplied by the influence of that fitnotype upon fitness in that environment (Fig 1B). Whereas the influence of each mutation upon each fitnotype is assumed constant across environments yet variable for each mutation, the weighted influence of that fitnotype within a given environment is constant across the mutations that affect it. First, this analysis revealed that just three or four fitnotypes were sufficient to explain a large portion of the variance in fitness even in disparate base environments. Second, upon changing the base environment within which perturbations were performed, they found that the fitnotypes recovered partially overlapped with those inferred for the original environment (Fig 1C and 1D).

At first blush, this outcome that just a few, major cellular phenotypic traits determine cellular fitness seems absurd. While it may be reasonable to expect that the performance of a single, definable biological ‘unit’ could be successfully abstracted as a small number of key traits representing the fitnotypes for that unit—e.g., for an enzyme: stability, activity, and binding affinity—the apparent over-simplification of treating a whole cell as just a few fitnotypes is jarring. Abundant data would say it is not so, that mutations can have many idiosyncratic patterns of pleiotropic effects. Indeed, comprehensive mutant libraries have uncovered that, while some genes have matching phenotypic profiles across environments, such as mutations impacting subunits of the same protein or enzymes of the same pathway, phenotypic patterns observed across test environments generally are on the order of a hundred distinct types [4]. With so many fitnotypes in those experiments, how can there possibly be just three or four fitnotypes here?

As clearly explained by Ghosh and colleagues, the key difference is their focus upon mutations arising during the initial stage of adaptation. The need to escape drift and fare well compared to other beneficial mutations arising simultaneously in these populations bias towards high fitness mutations as they rise to a reasonable frequency. While mutations in hundreds or thousands of genes may have been deleterious in a single environment [4], results from many evolution experiments suggest that there is only a small set of targets that can be substantially beneficial and establish across replicate populations [5]. This empirical finding is bolstered by theory. For example, within metabolism, Metabolic Control Analysis has illuminated this asymmetry due to the saturating effect of individual enzyme levels upon steady-state flux through the system [6]. This new analysis by Ghosh and colleagues [3] goes above and beyond prior work to show that even the relatively modest number of loci in which the beneficial mutations occurred funnel down into an even smaller number of fitnotypes.

More generally, this paper is an excellent case of using unsupervised clustering of interaction data to uncover the ‘structure’ of biological systems. This approach has proven quite successful previously, such as in the analysis of the effect of synergistic versus antagonistic interactions (i.e., epistasis) between pairs of enzyme knockout mutations upon growth rate which revealed sets of enzymes equivalent to known, named modules of metabolism [7]. Similarly, antibiotics could be clustered according to their target simply from whether they had consistent synergistic or antagonistic interactions with antibiotics from other such groups [8]. In the same manner, the fitnotypes described here were revealed *post hoc* via statistical analysis of the correlation structure. As such, in some sense they may be thought of as a ‘retrodiction’ rather than a prediction. On the other hand, a great advantage to applying fitnotype inference is that it is agnostic to known physiology. This contrasts with classical examples of ab initio prediction of evolution based on biochemical mechanism. While such work represents a wonderful synthesis, it has been limited to particular biological systems—like lactose transport and cleavage in a lactose-limited chemostat [9]—whose critical role in fitness in the specific, tested environment was known *a priori*.

What, then, from a mechanistic perspective, do these fitnotypes represent? One enticing possibility is that these represent key axes of cellular physiology that modulate large swaths of gene products. An example would be the growth laws that set the balance between protein sectors required for ribosomes, anabolism, catabolism, and house-keeping functions [10]. The simple formalism of coarse-grained models that relate growth to the optimal allocation of the proteome has been remarkably successful in capturing many cellular phenomena [10]. These growth laws arise from both critical proteins, such as alternate sigma factors, and key secondary metabolites, such as cAMP and (p)ppGpp [10]. Indeed, multiple evolution experiments have uncovered early beneficial mutations that impacted these over-arching conductors of the cell [11,12]. Future work that links cellular physiology to the conclusions from statistical patterns showing global epistasis [1,2] and various types of pleiotropy [13] will be critical. Given that recent work examining mutational interactions, rather than environmental ones like those explored here, also revealed relatively few phenotypic dimensions [14], perhaps there is hope for further synthesis of these currently disparate views of biological ‘structure’ and efforts can be directed towards understand their molecular basis.

It is daunting to consider that all the above examples used planktonic growth in liquid as the sole setting to consider fitness, when cells are confronted with so many more challenges in nature. In a wider set of test environments and mutations, it is likely that a much wider set of fitnotypes will emerge that reflect a fuller range of ecological or developmental processes, such as sporulation, motility, biofilm production, and more. Ghosh and colleagues [3] present an elegant approach that is a theoretical leap forward using statistics to grab a tiger by the tail and tease that “something” exists, for now just labelled as a fitnotype, that generates the predictable G x E x E correlations that we can use to approach the structure of cells.

In the song of life, there is a massive ‘keyboard’ of phenotypes that are influenced by mutation. Selection, however, does not appear to reward randomly pounding on many keys at once. The outcomes of initial adaptation to a particular environment are remarkably simpler, structured by a meagre number of fundamental phenotypes. This seems far less like Mozart or Beethoven and far more like how Harlan Howard famously defined a successful country song: just “three chords and the truth.”
